# Anti-quorum sensing potential of selenium nanoparticles against LasI/R, RhlI/R, and PQS/MvfR in *Pseudomonas aeruginosa*: a molecular docking approach

**DOI:** 10.3389/fmolb.2023.1203672

**Published:** 2023-08-10

**Authors:** Kanak Raj Kanak, Regina Sharmila Dass, Archana Pan

**Affiliations:** ^1^ Fungal Genetics and Mycotoxicology Laboratory, Department of Microbiology, School of Life Sciences, Pondicherry University (A Central University), Pondicherry, India; ^2^ Department of Bioinformatics, School of Life Sciences, Pondicherry University (A Central University), Pondicherry, India

**Keywords:** antimicrobial resistance, *in silico* drug design, molecular docking, microbial nanoparticle interaction, PatchDock, *Pseudomonas aeruginosa*, quorum sensing, selenium nanoparticles

## Abstract

*Pseudomonas aeruginosa* is an infectious pathogen which has the ability to cause primary and secondary contagions in the blood, lungs, and other body parts of immunosuppressed individuals, as well as community-acquired diseases, such as folliculitis, osteomyelitis, pneumonia, and others. This opportunistic bacterium displays drug resistance and regulates its pathogenicity via the quorum sensing (QS) mechanism, which includes the LasI/R, RhlI/R, and PQS/MvfR systems. Targeting the QS systems might be an excellent way to treat *P. aeruginosa* infections. Although a wide array of antibiotics, namely, newer penicillins, cephalosporins, and combination drugs are being used, the use of selenium nanoparticles (SeNPs) to cure *P. aeruginosa* infections is extremely rare as their mechanistic interactions are weakly understood, which results in carrying out this study. The present study demonstrates a computational approach of binding the interaction pattern between SeNPs and the QS signaling proteins in *P. aeruginosa*, utilizing multiple bioinformatics approaches. The computational investigation revealed that SeNPs were acutely ‘locked’ into the active region of the relevant proteins by the abundant residues in their surroundings. The PatchDock-based molecular docking analysis evidently indicated the strong and significant interaction between SeNPs and the catalytic cleft of LasI synthase (Phe105-Se = 2.7 Å and Thr121-Se = 3.8 Å), RhlI synthase (Leu102-Se = 3.7 Å and Val138-Se = 3.2 Å), transcriptional receptor protein LasR (Lys42-Se = 3.9 Å, Arg122-Se = 3.2 Å, and Glu124-Se = 3.9 Å), RhlR (Tyr43-Se = 2.9 Å, Tyr45-Se = 3.4 Å, and His61-Se = 3.5 Å), and MvfR (Leu208-Se = 3.2 Å and Arg209-Se = 4.0 Å). The production of acyl homoserine lactones (AHLs) was inhibited by the use of SeNPs, thereby preventing QS as well. Obstructing the binding affinity of transcriptional regulatory proteins may cause the suppression of LasR, RhlR, and MvfR systems to become inactive, thereby blocking the activation of QS-regulated virulence factors along with their associated gene expression. Our findings clearly showed that SeNPs have anti-QS properties against the established QS systems of *P. aeruginosa*, which strongly advocated that SeNPs might be a potent solution to tackle drug resistance and a viable alternative to conventional antibiotics along with being helpful in therapeutic development to cure *P. aeruginosa* infections.

## 1 Introduction

*Pseudomonas aeruginosa* is an example of an opportunity-driven infectious bacterium, which is Gram-negative in its natural state. This nosocomial bacterial infection is a prominent cause of a variety of illnesses. It spreads easily in healthcare settings. These germs are responsible for approximately 10% of nosocomial bacterial infections (Gellatly and Hancock, 2013). Immunocompromised patients are at a higher risk of contracting a nosocomial infection (Dye, 2014). *P. aeruginosa* targets individuals with burn wounds, chronic obstructive pulmonary disorder (COPD), cancer, cystic fibrosis, immunodeficiency, and severe infections requiring ventilation (such as COVID-19) and poses a particular challenge in intensive care units (ICUs) (Raman et al., 2018; Lansbury et al., 2020; Killough et al., 2022; Qin et al., 2022). *P. aeruginosa*, with its diverse set of virulence factors and innate adaptation to different conditions, is responsible for a slew of severe, possibly deadly acute and chronic infections, particularly in immunocompromised hosts, where fatality rates may exceed 40%. It is a lethal hazard to immunocompromised individuals. It is the most significant cause of bacteremia and sepsis in neutropenic cancer patients receiving chemotherapy and the predominant cause of hospital-acquired pneumonia and respiratory failure (Osmon et al., 2004; Matos et al., 2018; Killough et al., 2022; Wood et al., 2023). *P. aeruginosa* infections are also prevalent in surgical wounds, corneal, and diabetic ulcers. Additionally, there is a growing incidence of life-threatening *P. aeruginosa* infections in AIDS patients (Shepp et al., 1994; Wood et al., 2023).

*P. aeruginosa* adopts a biofilm mode of existence in which it evolves extreme persistency to both antibiotics and immune defense cascade, leading to multidrug resistance as a consequence of excessive selective pressure from uncontrolled consumption of conventional antibiotics (Su et al., 2010; Abinaya and Gayathri, 2019). *P. aeruginosa* is resistant to numerous antibiotics, such as aminoglycosides, quinolones, and lactams (Pang et al., 2019). In general, the strategies used by *P. aeruginosa* to resist antibiotics can be categorized as intrinsic, acquired, or adaptive resistance. Low outer membrane permeability, the expression of efflux pumps that eject antibiotics out of the cell, and the synthesis of antibiotic-inactivating enzymes comprise an intrinsic resistance approach of *P. aeruginosa*. *P. aeruginosa* can achieve acquired resistance through horizontal transmission of resistant genes or mutational alterations (Breidenstein et al., 2011). Adaptive resistance, on the other hand, is characterized by the growth of biofilms in the infected patients, which act as barriers limiting antibiotic penetration to the bacterial cells (Drenkard, 2003). This adaptive resistance enhances the survival of the bacterium against antibiotic assaults by transiently modifying gene and/or protein expression in response to environmental stimuli, and it can be reversed once the stimulus is removed (Sandoval-Motta and Aldana, 2016). Furthermore, within the biofilm, there is the development of multidrug-tolerant persister cells that can withstand antibiotic attacks, contributing to prolonged and persistent infections in affected patients. Among the various mechanisms, the formation of biofilms and the generation of persister cells are the most extensively studied aspects of adaptive resistance in *P. aeruginosa*, leading to persistent infections and unfavorable prognoses in affected individuals (Taylor et al., 2014).

The general mechanisms of biofilm-mediated resistance protecting bacteria from antibiotic attack involve the prevention of antibiotic penetration, altered microenvironment inducing slow growth of biofilm cells, and expression of an adaptive stress response permitting survival under harsh conditions that stimulate and alter the chemical microenvironment within the biofilm, which induces slow growth of bacteria, resulting as the reduction of antibiotic uptake and formation of multidrug-tolerant persister cells (Stewart, 2002; Balaban et al., 2013). Persister cells do not proliferate in the presence of antibiotics; however, they resume growth once the antibiotics are removed (Maisonneuve and Gerdes, 2014). Furthermore, environmental stimuli have an impact on the formation and proliferation of persister cells. Moreover, the quorum sensing (QS) signaling molecule acyl-homoserine lactone 3-OC12-HSL has been reported to greatly increase the number of persister cells in *P. aeruginosa* cultures (Moker et al., 2010; Mlynarcik and Kolar, 2017).

The pathogenic features of *P. aeruginosa* are modulated by a cell concentration-based process known as QS that utilizes QS molecules. The QS system is a bacterial intercellular signaling mechanism that is governed by the discharge of diffusible chemical compounds known as autoinducers (Reading and Sperandio, 2006; Banik et al., 2009) that are recognized by a receptor. These QS molecules regulate a number of detrimental behaviors in both the Gram-negative and positive bacteria, including an assembly of virulence factors and the biofilm generation (Davies, 2003). The bacterial regulatory system known as QS is involved in controlling an array of cellular cascading display, including the production of antibiotics, biofilm production, bioluminescence, competence, sporulation, and the secretion of virulence factors (Moghaddam et al., 2014). QS signaling is also employed by *P. aeruginosa* to control the expression level of genes and play an important role in its pathogenic activity (Smith and Iglewski, 2003).

Reportedly, *P. aeruginosa* possesses four interconnected QS signaling networks, namely, LasI/LasR, RhlI/RhlR, *Pseudomonas* quinolone signal (PQS)/MvfR, and IQS (integrated QS). Within these networks, LasI and RhlI synthase enzymes activate the Las and Rhl pathways, which are responsible for producing acyl homoserine lactone (AHL) signaling molecules, such as 3-oxododecanoyl-l-homoserine lactone (3-oxo-C12-HSL) and butyryl-l-homoserine lactone (C4-HSL), respectively (Lee and Zhang, 2015; Rampioni et al., 2016). These molecules, 3-oxo-C12-HSL and C4-HSL, bind and activate their respective transcription factors, LasR and RhlR, triggering biofilm formation and the expression of various virulence factors, such as elastase, proteases, pyocyanin, lectins, rhamnolipids, and toxins (Rutherford and Bassler, 2012). The initiation of the third QS system occurs by the transcriptional regulator MvfR (PqsR) by binding to 2-heptyl-3-hydroxy-4(1H)-quinolone, which is a PQS signaling molecule (Déziel et al., 2005), to promote biofilm formation. The PQS-MvfR system controls the production of the *Pseudomonas* quinolone signal (PQS) through the regulation of the pqsABCDE operon by the transcriptional regulator MvfR, also known as PqsR. These PQS signaling molecules work in the absence or dysfunction of LasR and activate the virulence genes (Lee and Zhang, 2015). Lee and co-workers (2013) suggested a novel QS molecule, namely, 2-(2-hydroxyl-phenyl)-thiazole-4-carbaldehyde, which constituted a fourth QS system, i.e., IQS (Lee et al., 2013). However, since its discovery, there has been debate over IQS, and the specific role of the IQS molecule in the QS system requires further exploration (Lee and Zhang, 2015; Lin and Cheng, 2019). We are excluding IQS from the current analysis due to the lacuna of reliable data about the process of IQS creation and their significance in the QS system (Cornelis, 2020). As a result, only the inhibitory potential of selenium nanoparticles (SeNPs) against the Las, Rhl, and PQS QS systems was studied in the present investigation and analysis.

*P. aeruginosa* manages their contained virulence by generating, perceiving, and reacting to autoinducers, which are extracellular signaling molecules that attach to a receptor (Rutherford and Bassler, 2012; Longo et al., 2013). Investigations concerning *P. aeruginosa*-based QS signaling molecules indicate that these autoinducers (AHLs) influence the synthesis of virulence factors and their relative consequences (Pearson et al., 1997; Van Delden and Iglewski, 1998; Pesci et al., 1999). When homo serine lactones and PQS autoinducers are present at optimal concentrations, they bind to specific receptor proteins, namely, LasR, RhlR, and MvfR, and stimulate the transcription of numerous genes responsible for producing various virulence factors, such as elastases, pyocyanin, rhamnolipids, and siderophores (Passador et al., 1993; Strateva and Mitov, 2011; Moradali et al., 2017). In *P. aeruginosa*, these QS signaling molecules are liable for infection; thus, proteins implicated in QS signaling could be serving as potential targets to develop antimicrobial agents. Although antibiotics are available to treat the illness, the combined effect of the QS complex causes infection; nevertheless, the antibiotic treatment is ineffectual since resistance develops in a short amount of time, making this bacterium a serious threat (Juan et al., 2010; Su et al., 2010; Ali et al., 2017; Abinaya and Gayathri, 2019). Reports suggested that a defect in QS systems renders *P. aeruginosa* less virulent and also results in the production of a flat biofilm that is susceptible to antibiotics (Jensen et al., 2007; Van Gennip et al., 2009). Therefore, disrupting these bacterial QS systems could be beneficial in the treatment of various diseases due to the fact that QS molecules are instrumental in controling virulence (Alvarez et al., 2012). Thus, inhibiting quorum sensing is regarded as a promising strategy for combating *P. aeruginosa* infections (Hurley et al., 2012). This approach can effectively prevent biofilm formation, diminish bacterial virulence, and carry a low risk of provoking bacterial resistance (Reuter et al., 2016). Moreover, this strategy exhibits a narrow spectrum, making it unlikely to inadvertently inhibit beneficial bacteria (Pang et al., 2019). As a result, a cost-effective and alternative therapy to treat *P. aeruginosa* infection is required. Moreover, the development of new antibiotics is very limited and time consuming. Thus, the development of novel therapeutic approaches to treat *P. aeruginosa* infections is highly desirable and has gained much attention in the past decade (Hurley et al., 2012; Chatterjee et al., 2016).

Nanoparticles could potentially interrupt QS molecules, allowing nanotechnology to provide a solution to this problem. Additionally, nanoparticles have the benefits of reducing toxicity, conquering resistance, and being more economical (Pal et al., 2007; Weir et al., 2008). Mühling and coworkers evidenced that native habitant bacteria have no resistance to nanoparticles (Mühling et al., 2009). As a result, several researchers have begun to use nanotechnology to generate advanced versions of antimicrobials, such as QS-targeted nano-inhibitors, in recent years. The approaches under this field could lead to benefits such as enhanced coating and dispersion of biofilms, increased solubility, ease of administration, and the maintenance of QS inhibitor activity (Nafee et al., 2014). In this regard, the production of bio-metallic nanoparticles is an emerging route as a potential, environmentally accessible, and nontoxic alternative to chemical or physical synthesis of nanoparticles. The antimicrobial activity of numerous nanoparticles has been reported in the past few years, including silver, gold, polymeric, and tin oxide nanoparticles, all of which have various modes of action (Samanta et al., 2017; Al-Shabib et al., 2018; Flockton et al., 2019). Only few scientific studies have demonstrated the inhibitory potential of SeNPs to QS-mediated virulence factors and that they have anti-biofilm function (Singh et al., 2017; Gómez-Gómez et al., 2019). Various accounts proclaiming NPs to be anti-QS agents exist, yet only a few studies have concentrated on the mechanistic approach and computational analysis of SeNPs to discover the possibility of them becoming anti-QS agents (Singh et al., 2017; Gómez-Gómez et al., 2019). Considering the significance of computational analysis and the scarcity of literature on the mechanics of SeNPs interacting with QS-controlled virulence genes, we set out to accomplish a few objectives: 1) computational investigation to check the potential of SeNPs as an anti-QS agent, 2) analysis of binding between SeNPs and QS-regulatory proteins, i.e., the LasI/RhlI-based AHL synthase, using the molecular docking approach, 3) to optimize the bonding between SeNPs and LasR/RhlR, a transcriptional regulatory protein in QS of *P. aeruginosa* using molecular docking, and 4) assessment of the residual-binding pattern between SeNPs and a PQS signaling receptor protein MvfR, using protein–ligand docking.

## 2 Materials and methods

### 2.1 Retrieval of the protein sequences and homology modeling

The crystallographic 3D structural design of the three key QS proteins LasI synthase, transcriptional activator protein LasR, and MvfR transcriptional protein with their respective PDB IDs 1RO5 (resolution = 2.3 Å), 2UV0 (chain E, resolution = 1.8 Å), and 4JVC (resolution = 2.5 Å) was retrieved from the Protein Data Bank (PDB; www.rcsb.org). However, the PDB did not contain the 3D crystal structures of two other QS-related proteins, i.e., RhlI synthase and RhlR. Therefore, homology modeling was carried out in order to model the 3D structures of each of these proteins. The RhlI synthase and RhlR amino acid sequences were obtained from the National Centre for Biotechnology Information (NCBI) (http://www.ncbi.nlm.nih.gov/) and stored as a FASTA file with the corresponding accession numbers P54291.2 and AAC44036.1, respectively, as the query sequence and employed for homology modeling. The tertiary structure of RhlI synthase and RhlR was predicted by homology modeling in the Robetta server (Raman et al., 2009; Song et al., 2013). Robetta (http://robetta.bakerlab.org) is a web-based platform that offers automated estimation of structure and analysis tools for inferring protein structural characteristics from genomic sequences. The service makes use of the first completely automated structure forecasting approach, which generates a model for a whole protein sequence irrespective of whether it overlays or not its sequence homology with protein(s) whose structures are already known. Robetta creates structural models of proteins by parsing the protein sequence into potential domains. Robetta protein modeling utilizes both comparative modeling, which is employed when a suitable template match is found, and *de novo* modeling, which is used when a suitable template match is not available. In this study, PSI-BLAST was employed to perform amino acid sequence alignment, and the top 20 hits with the highest identity were chosen as templates for further investigation.

### 2.2 Structure model evaluation and validation

The selected homology model was evaluated for its compatibility with different structural variables using reliable and comparable assessment tools, such as WHAT IF, PROCHECK, ERRAT, and VERIFY-3D. These tools were employed to assess and verify different aspects of the structural quality of the model. The final structure of the modeled protein was analyzed by the WHAT IF server (Vriend, 1990) followed by the SAVES server (http://nihserver.mbi.ucla.edu/SAVES/). The stereochemical accuracy with the overall structural geometry of the modeled protein was evaluated and confirmed using PROCHECK, a program that evaluates the stereochemical quality of protein structures (Laskowski et al., 1993). A structure evaluation server, VERIFY-3D (Lüthy et al., 1992), was utilized to evaluate the compatibility of the structure of the model and its corresponding amino acid residues, which assigned a structural class to the model considering its location and surroundings. In addition, ERRAT graphs were generated to further evaluate the compatibility of the model. Ramachandran plot statistics were analyzed using the online server RAMPAGE to evaluate the stability of the model. Furthermore, Ramachandran plot analysis was implemented to evaluate the complete stereochemical content of the protein and the amino acid residues in the permitted and disallowed regions.

### 2.3 Molecular dynamics simulation of the modeled proteins

The best-scored modeled tertiary structure of the RhlI synthase and RhlR obtained from the Robetta server was chosen for further study. Molecular dynamics simulations were conducted to assess the stability of the modeled protein using GROMACS 2019 with the GROMOS 54a7 force field. The simulations were carried out for a duration of 100 nanoseconds (ns) in a cubic box with a size of 1.0 nanometer (nm). The box was loaded with simple point charges, and four Na^+^ ions were introduced to neutralize the system. For neutralization, the solvent molecule was replaced with Na^+^ ions in the SPC/E water model. A periodic boundary condition was implemented in every possible direction. The predicted models underwent energy minimization with a maximum of 50,000 steps employing the conjugate gradient algorithm and steepest descent minimization. The minimization process terminated when the force was below 1000.0 kJ/mol/nm. Subsequently, each system was equilibrated for 1000 picoseconds (ps) under NVT and NPT ensemble with constant number of their respective particles, volume, temperature, and pressure employing a Berendsen thermostat. The partial mesh Ewald algorithm was used to estimate the electrostatic and van der Waals interactions, with the short-range neighbor list cut-off, short-range electrostatic cut-off, and short-range van der Waals cut-off all set to 1 nm. The particle mesh Ewald method was employed for ensemble generation with a cut-off of 10. To ensure a steady environment, temperature at 300 K and pressure coupling at 1 bar were employed, with coupling values set to 2 and 0.1 ps, respectively. The compressibility was set to 4.5 e^−5/bar^. The whole setup underwent equilibration for 500 and 100 ps using NVT and NPT ensembles, respectively. Last, the equilibrated system was simulated without constraints for 100 ns with a time step of 2 fs using the NPT ensemble to explore the structural dynamics of the modeled proteins. GROMACS utility packages, such as g_rms and g_rmsf, were used to compute root-mean-square deviation (RMSD) and root-mean-square fluctuation (RMSF) for the homology model trajectories at 100 ns. All graphs were created using the GRACE software (http://plasma-gate.weizmann.ac.il/Grace/). RMSD amid the main-chain atom of model proteins between pre-simulation and post-simulation was determined by superimposition using PyMol (version 4.6.0).

### 2.4 Preparation of the ligand molecule

#### 2.4.1 Preparation of the 3D structure of SeNPs

The structure of the SeNPs was extracted using the PubChem database (https://www.ncbi.nlm.nih.gov/pccompound) in the *.sdf* data file format. Additionally, the conversion of 2D to 3D structures was carried out by using MarvinSketch (ver.15.11.30) and saved as a *.pdb* data file.

### 2.5 Molecular docking analysis

#### 2.5.1 Prediction of the docking or active site of Las, Rhl, and PQS systems

The CASTp tool was employed to predict the binding sites of SeNPs for the generated LasI/R and RhlI/R models along with MvfR protein. The *.pdb* files were uploaded into the tool, and subsequent processing of these files enabled the selection of active binding sites for SeNP ligands.

#### 2.5.2 Molecular docking

Molecular docking was carried out using the rigid protein structures of the LasI synthase, LasR receptor, RhlI synthase, and RhlR receptor. Specifically, the interaction between SeNPs and LasI (AHL synthase), as well as LasR/MvfR (transcriptional receptor proteins), was examined separately using the PatchDock online program. The interactions of SeNPs with the AHL synthase LasI, LasR receptor, and AHL assess long-range electrostatic interactions. The synthase RhlI and RhlR receptor, along with PQS receptor MvfR, were examined and explored with the assistance of the PatchDock tool (Schneidman-Duhovny et al., 2005). The protein files were modified by removing water molecules along with their native ligand. Ligand files were prepared according to the specifications of the tool, and default docking parameters were maintained. PatchDock, a geometry-based molecular docking algorithm, which has been critically assessed by the CAPRI initiative, was employed. PatchDock detects the most efficient protein–ligand interacting partner by assessing the conformational compatibility of flexible molecular interfaces. The RMSD clustering value for this parameter was kept at 4 Å as the default parameter recommended by the tool. In the post-docking process, the docking geometric score, desolvation energy, and ligand-interacting amino acids of the QS regulator proteins were evaluated using the output of the result provided from the PatchDock service. The scores of the docked files were then selected based on their ranking, and the figures were visualized using PyMol 3D visualization software (DeLano, 2002).

## 3 Results and discussion

SeNPs have low toxicity and great biocompatibility. As a result, they have attracted a great deal of interest with adoption in the biomedicine and food business organizations (Tran et al., 2015; Gunti et al., 2019). Selenium is an essential component in the production of selenoproteins, which include important antioxidants, such as deiodinase, glutathione peroxidase, and thioredoxin reductase (Rotruck et al., 1973). This is considered an essentially required trace element for the upkeep of human health, and the recommended amount of selenium in an adult’s daily dietary supplement ranges from 40 to 400 μg (Arthur, 1991; Rayman, 2005). Consequentially, the use of SeNPs is extremely acceptable and recommended in pharmaceutical and food industries. Among its many beneficial properties, SeNPs are an important metalloid trace element that possesses notable antimicrobial, antioxidant, and anticarcinogenic potential. Furthermore, previous studies have shown that SeNPs synthesized through biological means exhibit anti-QS and anti-biofilm properties against numerous pathogens (Singh et al., 2017). The research team also demonstrated the antibiofilm potential of selenium nanovectors and showed masked interest in the interaction of SeNPs with the LasR QS protein. However, there needs to be more *in silico* findings on the association of SeNPs with Las, Rhl, and PQS regulatory proteins associated with the *P. aeruginosa* QS system. Therefore, we adopted an *in silico* method to conduct exploratory research into the QS-modulatory mechanism of SeNPs. It is generally agreed upon that *P. aeruginosa* makes use of QS in the form of the synthesis of AHLs and Qunilone molecules with the aim to regulate an array of virulence-associated genes (Hodgkinson et al., 2012). Therefore, it is believed that SeNPs can disrupt AHL and quinolone-based QS mechanisms, thereby reducing virulence in *P. aeruginosa*. This makes SeNPs a promising candidate for novel therapeutic agents that can effectively combat QS-controlled infections. Consequently, we conducted computational exploratory research into the QS-controlled functioning of SeNPs.

In *P. aeruginosa*, the production of 3-oxo-C12-HSL and C4-HSL is controlled by LasI and RhlI synthases, respectively. These signaling molecules then activate the transcriptional activators LasR and RhlR, resulting in the induction of genes responsible for virulence. In the absence or inactivation of LasR, the MvfR transcriptional regulator, the receptor of the PQS QS signal, regulates the RhlI expression and LasR-controlled LasI genes that activate the RhlI/R system and direct biological signal transduction to virulence gene expression toward QS activity (Moradali et al., 2017). Thus, SeNPs were targeted against the Las signaling system, Rhl signaling cascade along with PQS signaling machinery. To gain insights into the inhibitory mechanisms of SeNPs against AHL synthases (LasI/RhlI) and transcriptional regulatory receptor proteins (LasR/RhlR and MvfR), the current study conducted molecular docking analyses to predict the potential interacting positions of SeNPs on these proteins.

**FIGURE 1:**
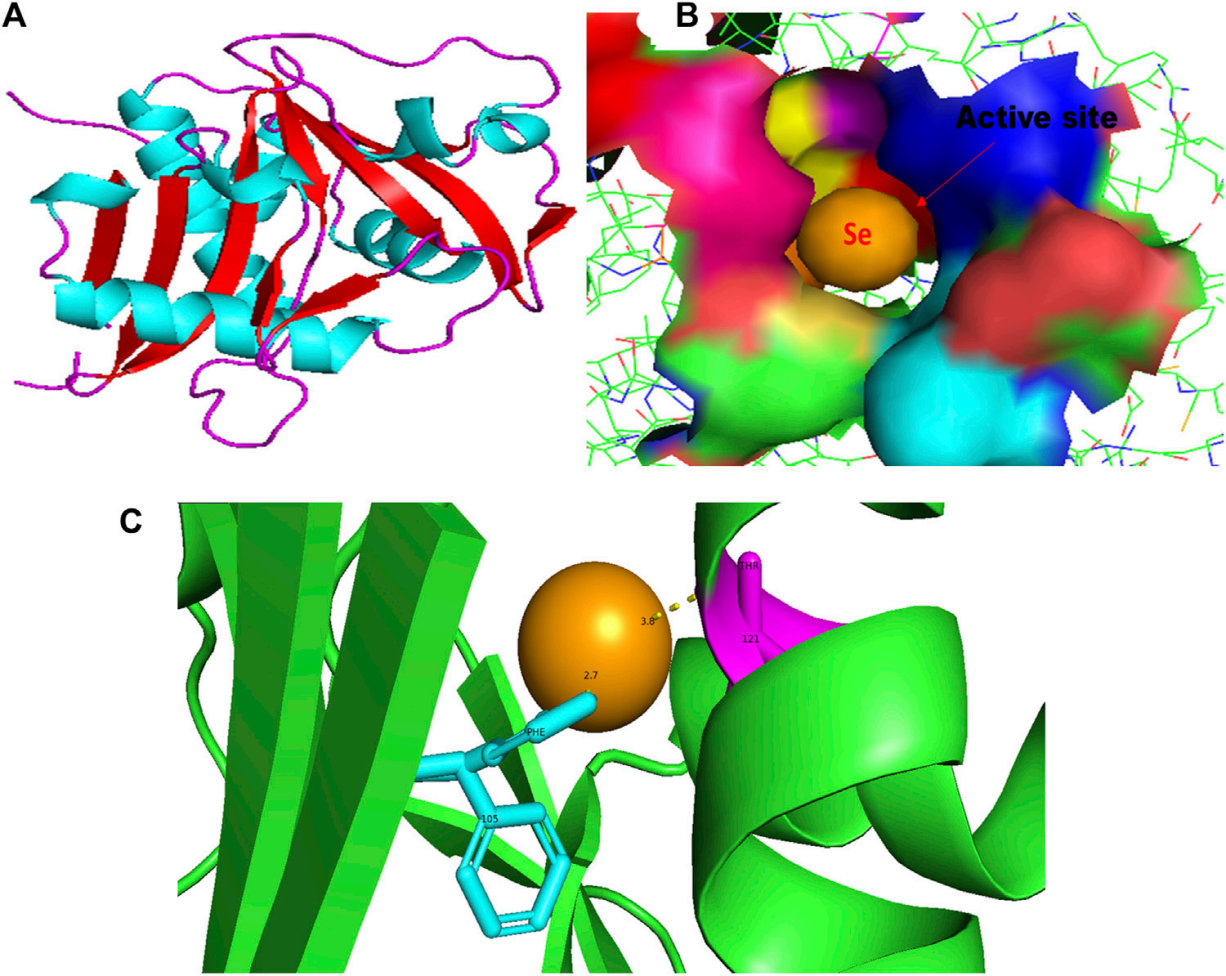
PyMol viewer showed the active location of PatchDock interactions of SeNPs with LasI. **(A)** 3D structure of AHL synthase LasI; **(B)** a close-up of the attachment of SeNPs to the LasI’s catalytic site, with bound Se depicted as a sphere; and **(C)** LasI amino acid residues (Phe105 and Thr121) that interact with SeNPs.

**FIGURE 2:**
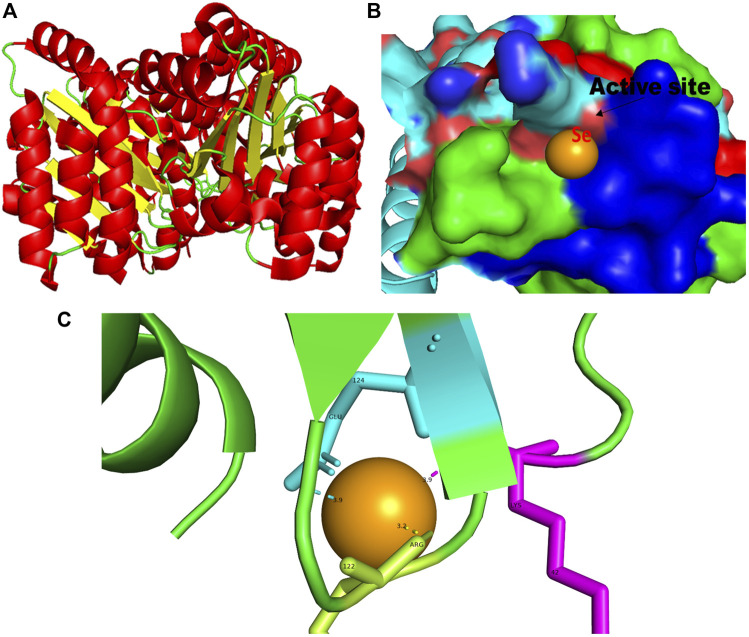
PyMol viewer showed the active location of PatchDock interactions of SeNPs with LasR. **(A)** 3D structure of transcriptional activator protein LasR; **(B)** a close-up of the attachment of SeNPs to LasR’s catalytic site, with bound Se depicted as a sphere; and **(C)** LasR amino acid residues (Lys42, Arg122, and Glu124) that interact with SeNPs.

**FIGURE 3:**
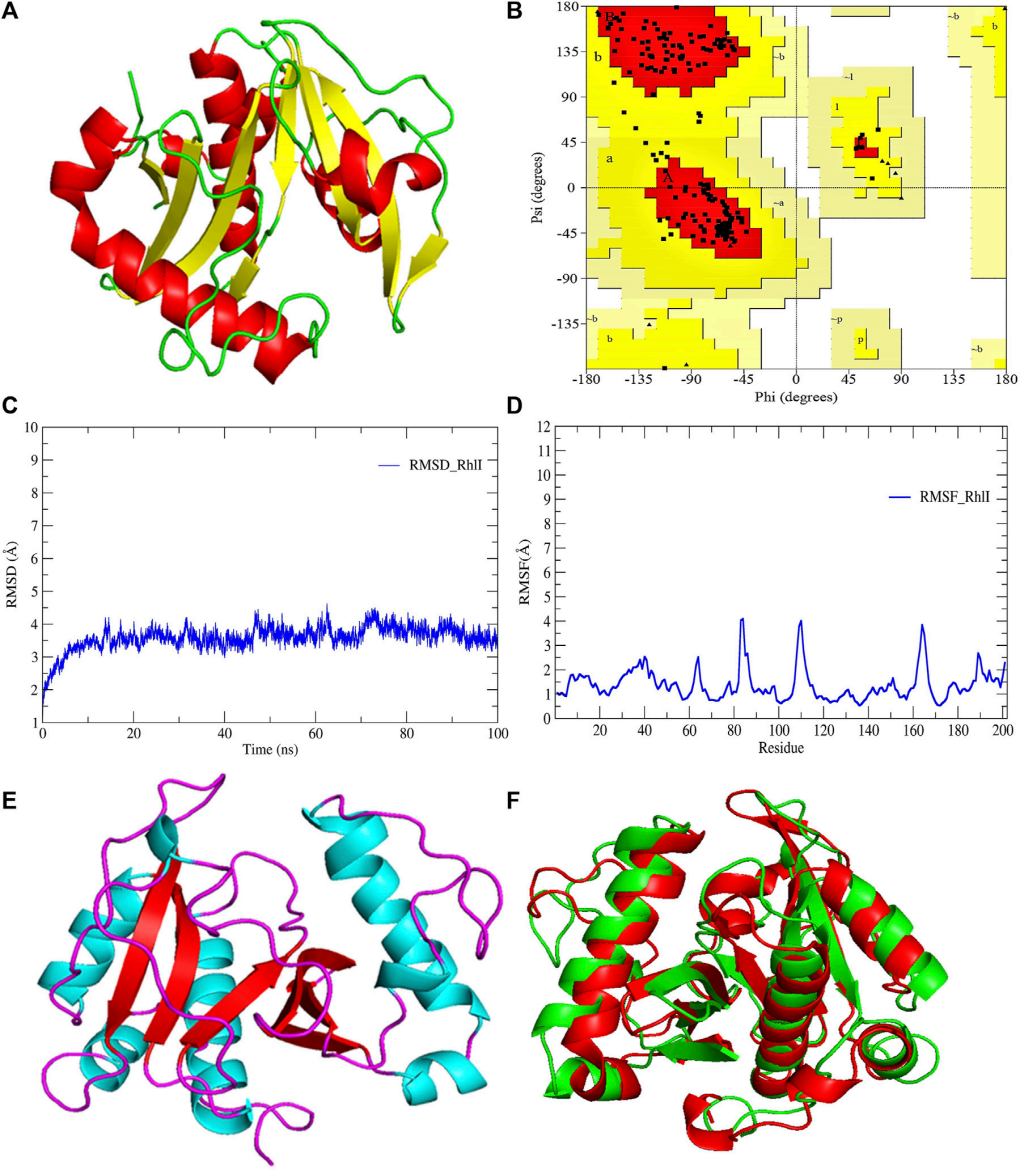
**(A)** 3D-modeled structure of AHL synthase RhlI at 0 ns using the Robetta server; **(B)** conformation validation of RhlI using RAMPAGE; **(C)** RMSD plot; **(D)** RMSF plot; **(E)** 3D-modeled structure of AHL synthase RhlI at 100 ns; and **(F)** superimposition of the 3D structure of modeled RhlI of 0 ns and 100 ns. Structure stability of modeled RhlI was explored by molecular dynamic simulation using GROMACS.

**FIGURE 4:**
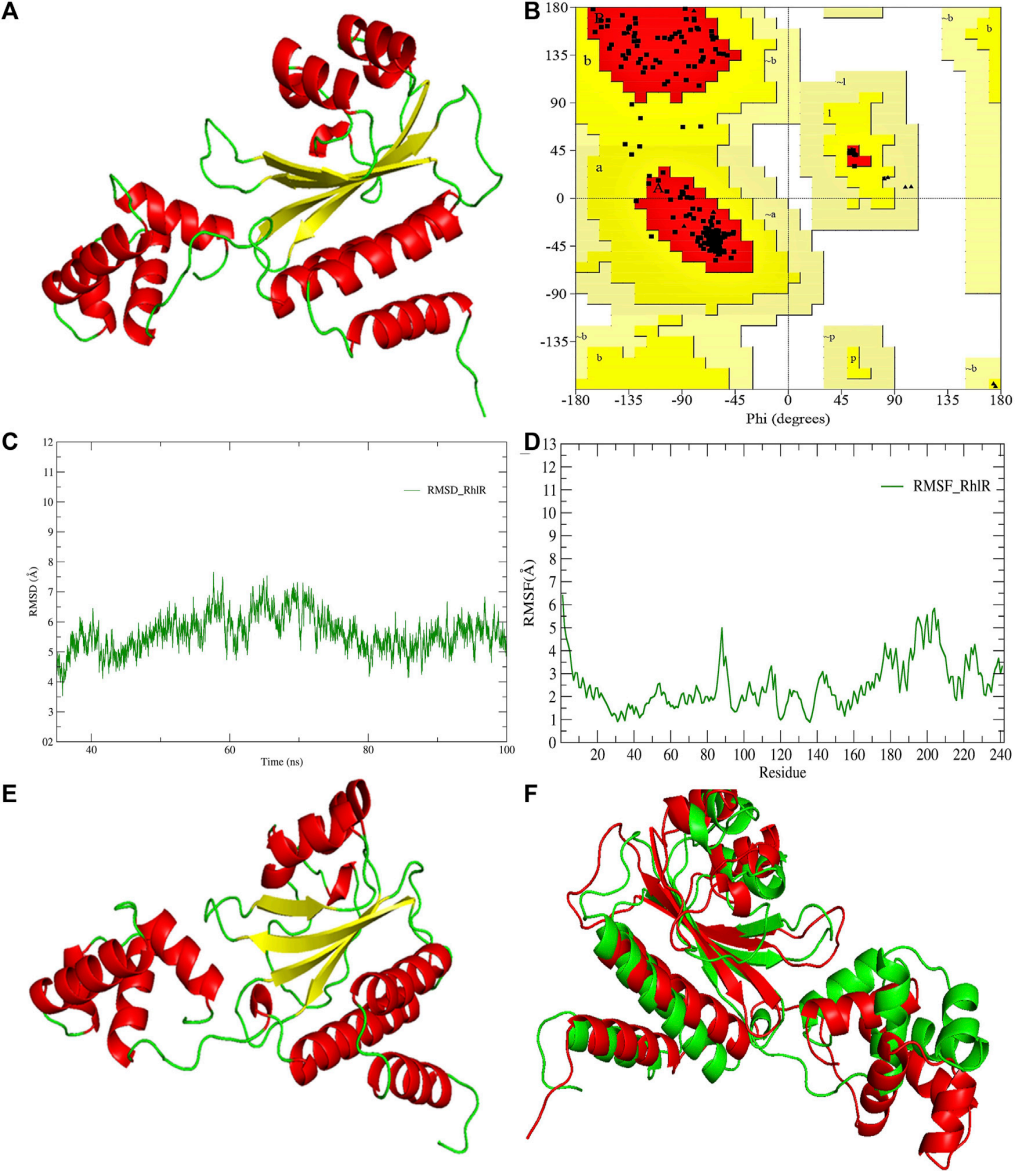
**(A)** 3D-modeled structure of AHL synthase RhlR at 0 ns using the Robetta server; **(B)** conformation validation of RhlR using RAMPAGE; **(C)** RMSD plot; **(D)** RMSF plot; **(E)** 3D-modeled structure of AHL synthase RhlR at 100 ns; and **(F)** superimposition of the 3D structure of modeled RhlR of 0 ns and 100 ns. Structure stability of modeled RhlR was explored by molecular dynamic simulation using GROMACS.

Consequently, we extracted the 3D crystal structures from the PDB database using the PDB IDs of 1RO5, 2UV0, and 4JVC, respectively, of the LasI synthase (Figure 1A), the transcriptional activator protein LasR (Figure 2A), and the MvfR transcriptional protein (Figure 9A). However, since no 3D crystal structure was available for the RhlI synthase and its regulatory protein in the PDB, we utilized the homology modeling method using the Robetta server to generate the 3D structure of the RhlI synthase (Figure 3A) and RhlR (Figure 4A). Homology modeling is a widely used technique for comparative modeling of protein structures when the PDB database lacks complete crystal structures. This is especially useful as protein sequences were generally more conserved than DNA sequences. Once a model protein has been generated, various structure validation programs are available to assess its geometrical conformations and stereochemical quality. Calculations for the Ramachandran plot were carried out with the help of the programs, such as PROCHECK, ERRAT, VERIFY-3D, and WHAT IF servers, followed by the SAVES server (Table 1).

**TABLE 1:** Structural validation of modeled proteins.

PROTEIN	VERIFY 3D	ERRAT	WHATCHECK	RAMACHANDRAN PLOT
RhlI	93.03% of the residues have averaged 3D-1D score≥0.2	Overall quality factor	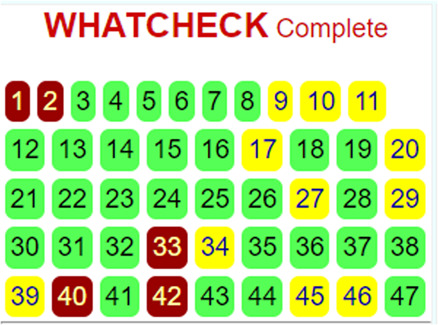	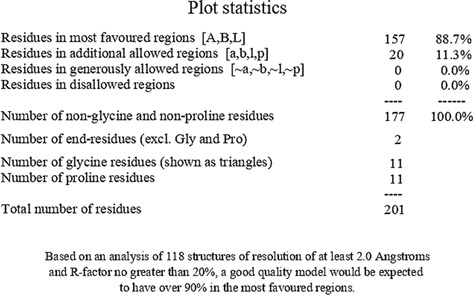
	**Pass**	**93.2292**		
RhlR	97.93% of the residues have averaged 3D-1D score ≥ 0.2	Overall quality factor	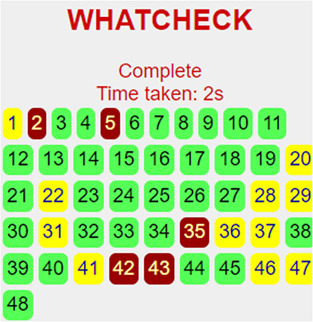	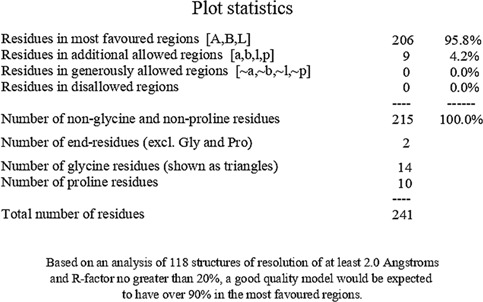
	**Pass**	**95.279**		

Model validation of the RhlI synthase and RhlR conformation was performed using the phi (Φ) and psi (Ψ) score analyses in a Ramachandran plot (Hollingsworth and Karplus, 2010). The modeled structure prediction depends on the highest sequence identity and coverage of the target sequence with the available template for model building. In the current study, the Ramachandran plot of the modeled RhlI synthase (Figure 3B) revealed that the number of non-glycine and non-proline residues is 177. Out of this, 157 (88.7%) were in the most favored regions along with 20 residues (11.3%) in the allowed regions and none in the outlier region. The allotment of main-chain torsion angle phi and psi evidently illustrated that the bulk of the amino acids are in a phi–psi distribution more or less reliable with right-handed alpha helices. These results imply that the stereochemical properties and quality of the model structure of the RhlI protein are quite suitable. Although previous studies (Laskowski et al., 1993) suggested that a good-quality model is expected to have a score over 90%, recent research (Williams and Cloete, 2022) demonstrated that the modeled structure is reliable if >80% of the residues are in the most favored region, followed by additional allowed regions for further residues (Williams and Cloete, 2022). This is very much in support of our model quality. In addition, if the model contains none of the residues in a disallowed region with the aforementioned conditions, then it is considered as a good-quality model. Scientific studies which report that a good-quality model having less than 90% in the most favored region are also available (Han et al., 2019; Chen et al., 2022; Williams and Cloete, 2022). Likewise, for RhlR, the Ramachandran plot revealed that 95.8% of the residues were in the preferred region, with 4.2% in the allowed region and none in the aberrant region (Figure 4B). The modeled tertiary structures of RhlI synthase and RhlR were subjected to investigate the stability of the modeled proteins. For determining the stability of a protein and its complexes, analysis of molecular dynamics simulations is indispensable. So, a molecular dynamics simulation for 100 ns was carried out using GROMACS 2019 and the GROMOS 54a7 force field.

**FIGURE 5:**
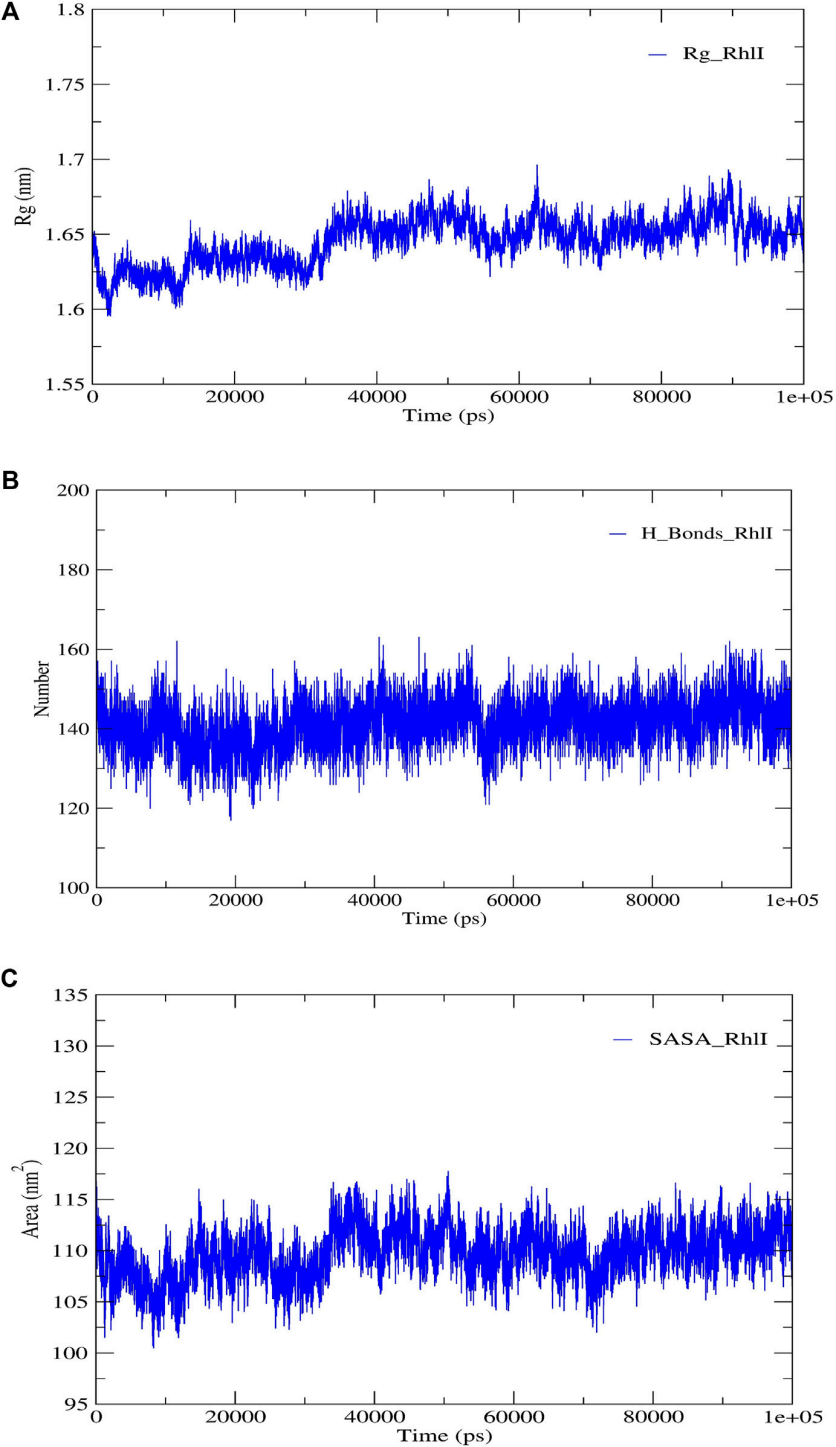
Structural validation of the modeled protein RhlI; **(A)** radius of gyration (Rg), **(B)** hydrogen bonds (H-bonds), and **(C)** solvent accessible surface area (SASA).

**FIGURE 6:**
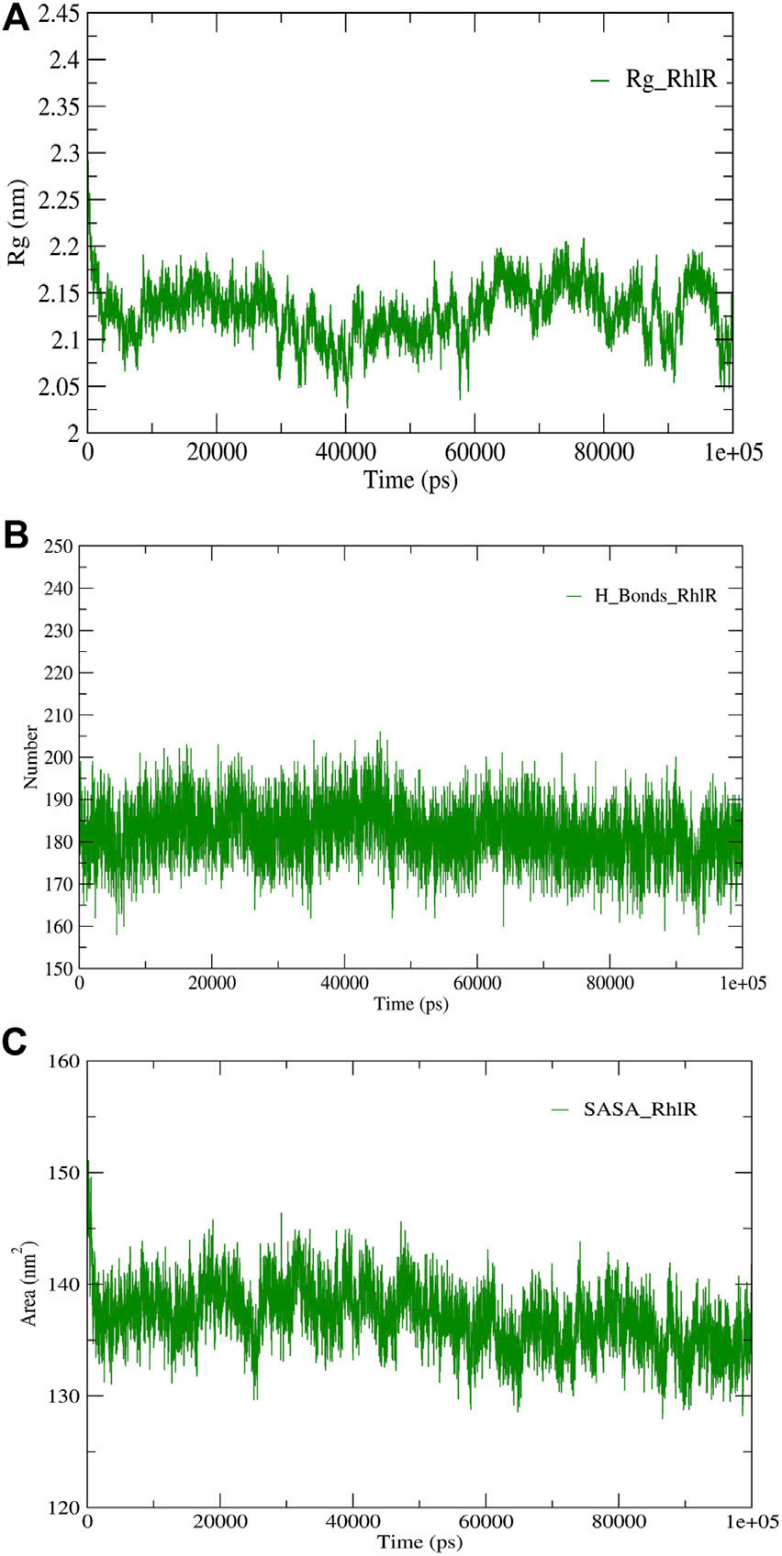
Structural validation of the modeled protein RhlR; **(A)** radius of gyration (Rg), **(B)** hydrogen bonds (H-bonds), and **(C)** solvent accessible surface area (SASA).

RMSD and RMSF were computed applying GRACE software for both the proteins RhlI and RhlR to understand their stability levels and the degree of flexibility over the amino acid residues in order to understand homology model trajectories generated at 100 ns (Figures 3C, D; Figures 4C, D). Our experimental settings demonstrated the protein’s stability over a brief period of time. The RMSD and RMSF calculations were evaluated and found to be under the threshold limit. The majority of amino acid residues in both proteins had lesser RMSF profiles, which may account for the inflexibility of the proteins (Figures 5, 6). Despite certain deviations observed in the result, the RMSF values of these residues in RhlI and RhlR stayed below 4.0 Å and 6.0 Å, respectively, Figures 3D, 4D. The RMSD values for the RhlI and RhlR proteins were around 3.8 Å and 5.8 Å, respectively, as shown in Figure 3C and Figure 4C.

Pre- and post-molecular dynamics simulation structure superimpositions have been performed using PyMol. The final snapshot from the trajectory of molecular dynamics at 100ns was superimposed over the modeled protein of 0 ns (Figures 3E, F; Figures 4E, F), and it revealed that the post-molecular dynamics simulated structure (100 ns) was more stable and had the same characteristics as the modeled structure (0 ns). As depicted in Figure 3F and Figure 4F, this stability of the protein can be attributed to the various residues and interactions present on its side chain. Notably, there was only a slight fluctuation observed in the side chain, and a small flexible region was identified. This may be due to a lack of contact or the presence of flexible loops.

The use of molecular docking, a powerful computational approach, has been employed to model the atomic-level interactions between small molecules and proteins to elucidate their behavior within binding sites. The functional grooves or pockets responsible for identifying the protein’s function are referred to as “active sites.” Although the active site typically constitutes only 10%–20% of the total protein, it catalyzes the entire enzymatic reaction. As a result, identifying a protein’s active or binding site is critical for understanding its functional behaviors. Hence, the targeted QS molecule docking/active site prediction was performed using CASTp (Table 2) for molecular docking studies.

**TABLE 2:** Active-binding sites of QS proteins.

Protein name	Amino acid positions in the active site
LasI	Val (26, 143, 148), **Phe** (27, 84, **105**, 117), Arg (30, 104), Trp (33, 69), Met (79), Glu (106), Leu (102), Ser (103), Ala (106), Ile (107), and **Thr** (**121**, 144, 145)
LasR	Phe (07, 167), Leu (08, 10, 125, 159, 165), **Glu** (11, **124**, 168), **Arg** (12, **122**), Ser (13, 14, 44, 161), Gly (15, 120, 123,164), Trp (19), Pro (41), **Lys (42)**, Asp (43), His (119), Ala (121, 163), and Gln (160)
RhlI	**Val** (27, 135, **138**, 163), Phe (28, 120, 173), **Leu** (32, 80, 81, 88, **102**, 124, 168), Trp (34), Glu (101, 166), Ser (103, 123), Arg (104), Tyr (105), Ala (107, 109, 110,137), Asp (111), Thr (139, 140), Gln (161), Lys (164), and Gly (165)
RhlR	Gln (25), Phe (28), Ala (29, 44), Glu (32), **Tyr (43, 45), His (61),** Gly (62), and Thr (63)
MvfR	Ala (102, 168, 237), Pro (129, 210, 238), Ile (149, 186, 189, 195, 236, 263), Thr (166, 265), Lys (167), Val (170, 211), Gly (194), Ser (196, 255), **Leu** (197, 207, **208**, 254), Asn (206), **Arg (209)**, Phe (221), Met (224), Trp (234), and Tyr (258)

**FIGURE 7:**
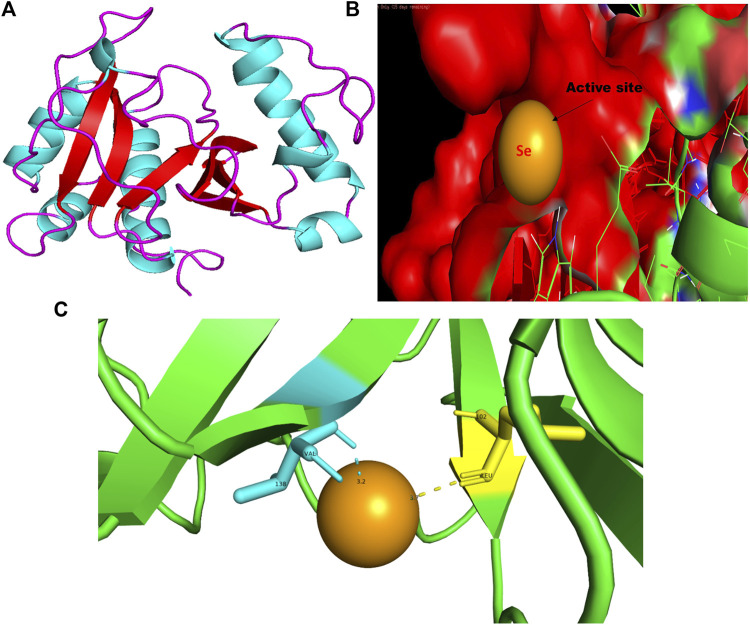
PyMol viewer showed the active location of PatchDock interactions of SeNPs with RhlI. **(A)** 3D-modeled structure of AHL synthase RhlI at 100 ns using the Robetta server; **(B)** a close-up of the attachment of SeNPs to RhlI’s catalytic site, with bound Se depicted as a sphere; and **(C)** RhlI amino acids residues (Leu102 and Val138) that interact with SeNPs.

**FIGURE 8:**
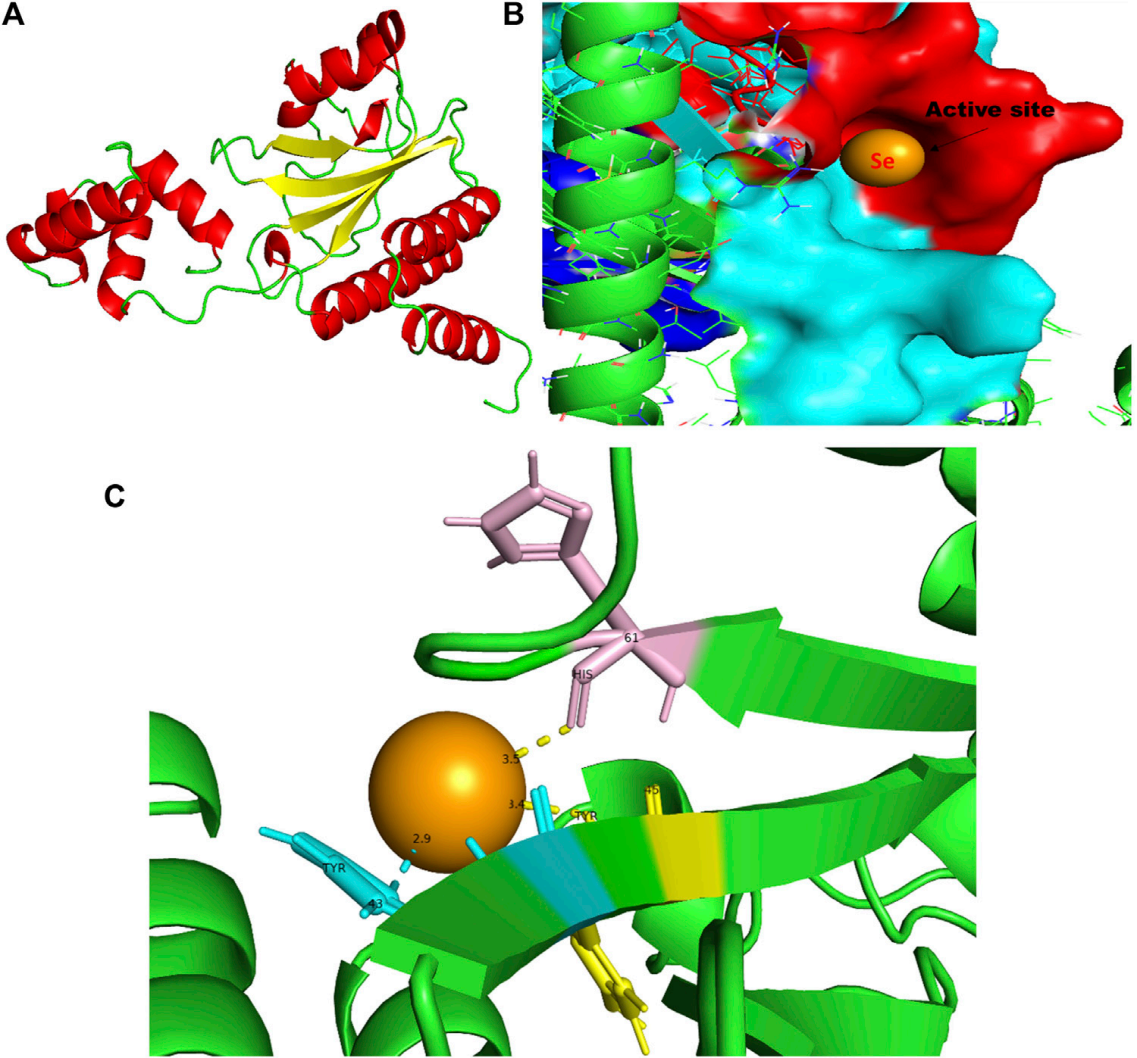
PyMol viewer showed the active location of PatchDock interactions of SeNPs with RhlR. **(A)** 3D modeled structure of AHL synthase RhlR at 100 ns using the Robetta server; **(B)** a close-up of the attachment of SeNPs to the RhlR’s catalytic site, with bound Se depicted as a sphere; **(C)** RhlR amino acids residues (Tyr43, Tyr45, and His61) that interact with SeNPs.

**FIGURE 9:**
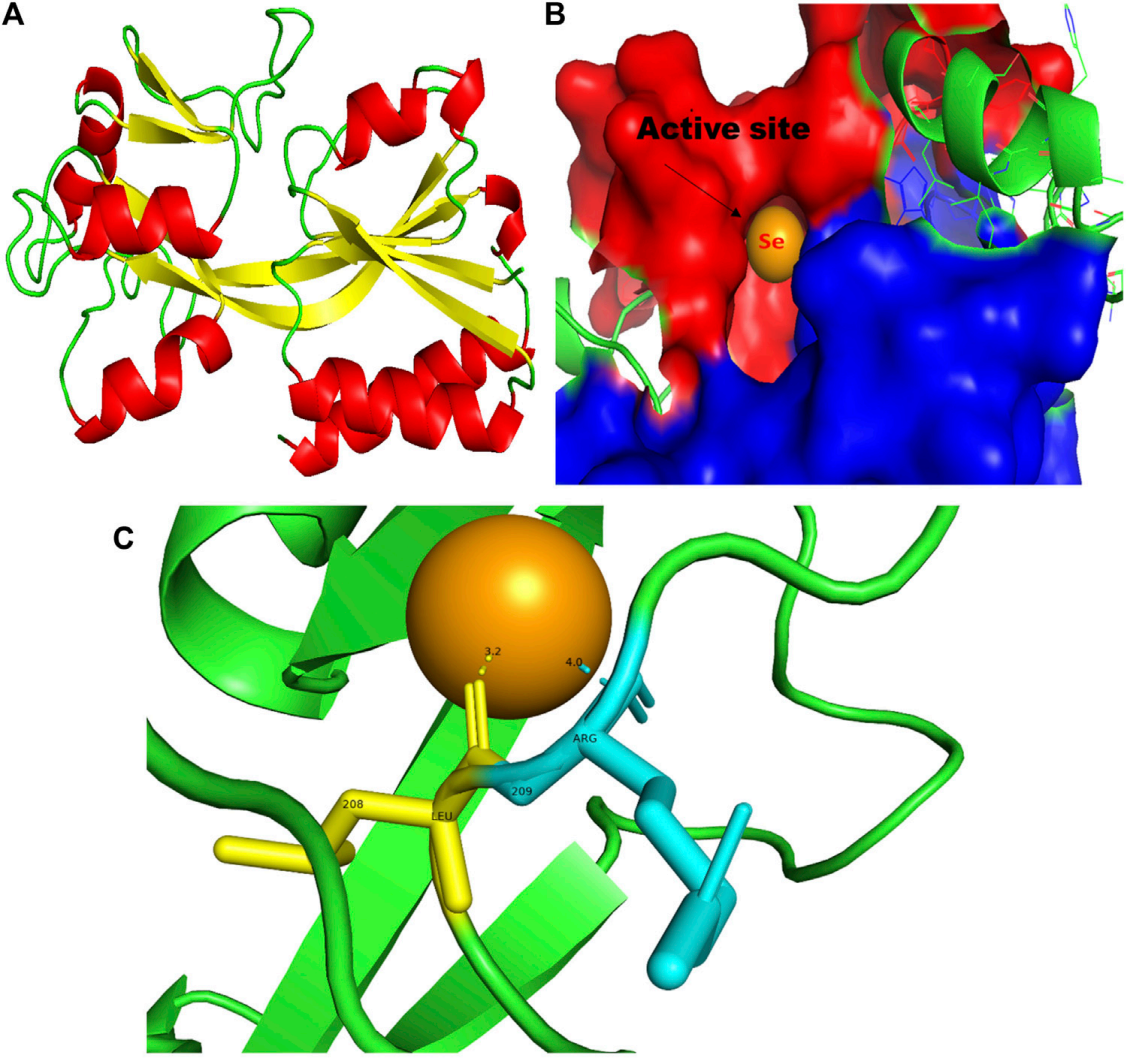
PyMol viewer showed the active location of PatchDock interactions of SeNPs with MvfR. **(A)** 3D structure of the transcription regulatory protein MvfR; **(B)** a close-up of the attachment of SeNPs to the MvfR’s catalytic site, with bound Se depicted as a sphere; and **(C)** MvfR amino acid residues (Leu208 and Arg209) that interact with SeNPs.

In this study, using a PatchDock-based molecular docking approach, it was found that SeNPs were successfully docked into the AHL synthases LasI (Figure 1B) and RhlI (Figure 7B), as well as the regulatory activator proteins LasR (Figure 2B) and RhlR (Figure 8B), and the transcriptional protein MvfR (Figure 9B). The docked structures after docking between the ligand and the respective interacting residues of QS proteins were displayed here using PyMol. On the basis of these docking positions, we speculated that SeNPs became ‘locked’ into the active site of specific proteins by their surrounding amino acid residues (Figures 1C, 2C, 7C, 8C, 9C). Table 3 displays the QS regulator proteins’ docking geometric score, atomic contact energy (desolvation energy), and ligand-interacting residues. There have been reports in the past showing the effectiveness of SeNPs as anti-QS and anti-biofilm agents against various pathogenic bacteria. However, there was a lack of *in silico* understanding on the interaction with bacterial QS regulator proteins. Thus, in the present study, computational docking of SeNPs with QS proteins (LasI, LasR, RhlI, RhlR, and MvfR) in the QS system of *P. aeruginosa* was performed.

**TABLE 3:** Docking result of the inhibitory potential of SeNPs with QS proteins.

Protein	Ligand	Binding score	Area	Atomic contact energy (kcal mol^-1^)	Name of interacting amino acids at active sites of proteins	
					Amino acid residues	Bond length (Å)
LasI	SeNPs	808	95.30	−55.23	Phe (105)	2.7
					Thr (121)	3.8
LasR	SeNPs	632	71.50	−54.30	Lys (42)	3.9
					Arg (122)	3.2
					Glu (124)	3.9
RhlI	SeNPs	712	87.50	−56.53	Leu (102)	3.7
					Val (138)	3.2
RhlR	SeNPs	720	77.90	−75.28	Tyr (43)	2.9
					Tyr (45)	3.4
					His (61)	3.5
MvfR	SeNPs	724	79.80	−65.03	Leu (208)	3.2
					Arg (209)	4.0

### 3.1 LasI

An essential component of the QS system in *P. aeruginosa* is the monomeric signaling protein, LasI synthase, which regulates the activity of virulence factors in the respective bacteria. This protein shows interaction with SeNPs. According to the results of molecular docking examination, Se^(0)^ binds to the catalytic residues phenylalanine and threonine of LasI synthase (Phe105-Se = 2.7 Å and Thr121-Se = 3.8 Å; Figure 1C). Therefore, the outcomes of a molecular docking of SeNPs with LasI determine that strong interactions and promising binding effectiveness at the active site of LasI have led to the formation of a more stable complex with a desolvation energy of −55.23 kcal mol^-1^. It displayed hydrogen bond interactions with Phe105 (2.7 Å) and Thr121 (3.8 Å) protein residues with a docking score of 808. In accordance with our study, the molecular interaction of nanoparticles has been reported in a few studies. Shah and their team also noted an electrostatic interaction of the Asp73 residue of LasI protein with silver nanoparticles (AgNPs) after docking analysis (Shah et al., 2019), which was similarly reported by Ali et al. (2017). Mishra and Mishra (2021) opined that copper nanoparticles (CuNPs) interact with amino acids Ser103, Leu102, Glu101, and Met79 in the LasI active site. Their study revealed that the formation of a stable protein–ligand complex of LasI and CuNP has a binding energy of −4.32 kcal mol^-1^ (Mishra and Mishra, 2021). In the current study, molecular docking analysis demonstrated SeNPs’ promising binding efficacy at the active site of LasI. The desolvation energy of the SeNP-LasI complex was notably higher (−55.23 kcal mol^-1^) compared to the well-known QSI furanone C30 (−2.39 kcal mol^-1^) and the natural ligand gingerol (−2.55 kcal mol^-1^) mentioned in other scientific reports (Shah et al., 2019; Mishra and Mishra, 2021). This finding strongly suggested that SeNPs could potentially contribute to the inhibition of AHL production.

### 3.2 LasR

LasR (PDB ID: 2UV0) is a transcriptional activator protein that spans 175 amino acids. It functions by binding to the autoinducer produced by the Las synthase protein. As a transcriptional activator, LasR plays a crucial role in activating multiple genes associated with virulence (Bottomley et al., 2007). Notably, the Las system acts as the primary controller of QS and triggers the countenance of both Rhl and Pqs signaling pathways in *P. aeruginosa* (Rathinam et al., 2017). Docking studies of SeNPs (Figure 2C) show a hydrogen bond interaction with arginine and a hydrophobic interaction with the amino acid lysine and glutamate residues of the LasR receptor (Arg122-Se = 3.2 Å, Lys42-Se = 3.9 Å, and Glu124-Se = 3.9 Å; Figure 2C) active site and make the bond with residues of the protein. The desolvation energy of the SeNPs was −54.3 kcal mol^−1^ with a docked score of 632, demonstrating that SeNP and LasR have effective and stable binds. Shah and co-workers (2019) reported AgNP production using the aqueous leaf extract of *Piper betle* and reported the electrostatic interaction with the amino acid Asp73 in the LasR active site (Shah et al., 2019), which was found to be in support of our study. A similar finding also has been reported by Vyshnava et al. (2016). In another report, it was stated that CuNP interacts with amino acid glutamic acid in the LasR active site displaying a significant binding energy −6.78 kcal mol^-1^ (Mishra and Mishra, 2021). The desolvation energy of SeNP (−54.3 kcal mol^-1^) was notably higher than the reported known QS inhibitor (QSI) furanone C30 (−5.44 kcal mol^-1^) (Shah et al., 2019), suggesting effective and steady interactions of SeNP and LasR. Therefore, this discovery indicated that the silencing of the transcriptional regulator LasR activity can be ascribed to the anti-QS activity of SeNPs along with their anti-biofilm function.

### 3.3 RhlI

The RhlI synthase is responsible for the production of N-butyryl-L-homoserine lactone, also known as C4-HSL. When C4-HSL binds to its cellular receptor RhlR, the virulence genes in *P. aeruginosa* are activated (Rutherford and Bassler, 2012). As a result of PatchDock, SeNP shows appreciable atomic contact energy (desolvation energy), −56.53 kcal mol^-1^, and a binding score of 712 with the protein residues, leucine and valine, in the active site of the RhlI synthase (Leu102-Se = 3.7 Å and Val138-Se = 3.2 Å; Figure 7C). In support of our study, Mishra and co-workers (2021) stipulated the similar interaction between known QS inhibitor gingerol and the amino acid Leu102 and Val138 of RhlI-binding pocket with binding energy −4.19 kcal mol^-1^ (Mishra and Mishra, 2021). Likewise, they also reported that CuNP interacted with amino acid glutamic acid and forms strong complex with RhlI with binding energy −4.43 kcal mol^-1^. These findings suggested that SeNP bonded strongly with RhlI. These results indicated that the suppression of transcriptional regulator RhlI endorsed the anti-QS activity of SeNPs.

### 3.4 RhlR

RhlR functions as a cellular receptor for AHL synthesized by the RhlI synthase, activating genes essential for virulence. The protein RhlR shows good binding with atomic contact energy −75.28 kcal mol^-1^ and a binding score (720) with SeNPs. SeNPs display hydrogen bond interactions with tyrosine and histidine residues of the RhlR receptor active site (Tyr43-Se = 2.9 Å, Tyr45-Se = 3.4 Å, and His61-Se = 3.5 Å; Figure 8C). This finding suggested that SeNP bonded strongly with RhlR. These results indicated that the suppression of transcriptional regulator RhlR endorsed the anti-QS activity of SeNPs. Mishra and team stipulated the interaction between the known QS inhibitor gingerol and the amino acids Phe, Arg, Ser, Val, and Ile of the RhlR-binding pocket with binding energy −1.82 kcal mol^-1^. The gingerol-interacting residues are in the active site line predicted in the current study. Likewise, they reported that CuNP interacts with amino acid glutamic acid and forms a strong complex with RhlI with binding energy −4.44 kcal mol^-1^ (Mishra and Mishra, 2021).

### 3.5 MvfR

In addition to LasI/LasR, PQS (*Pseudomonas* quinolone signal) is an additional QS system in *P. aeruginosa* that plays a significant role in biofilm formation (Dubern and Diggle, 2008). The synthesis of PQS molecules is controlled by the pqsABCDE operon through a transcriptional regulator protein called MvfR (PqsR). This protein also regulates the secretion of pyocyanin and rhamnolipid, as well as the growth of biofilm. Thus, targeting the inhibition of PQS is considered a promising strategy for attenuating pathogenic bacteria, particularly in biofilm-related infections (Rampioni et al., 2016; Parai et al., 2018). The PatchDock result shows SeNPs’ interaction with MvfR and forms the hydrogen bond with leucine and a hydrophobic interaction with arginine residues of the MvfR transcriptional protein (Leu208-Se = 3.2 Å and Arg209-Se = 4.0 Å; Figure 9C). The active site residues of MvfR exhibited a stable interaction with SeNPs, displaying a higher desolvation energy (−65.03 kcal mol^-1^) compared to QSI furanone C30 (−41.57 kcal mol^-1^), with a binding score of 724 (Table 3). This result is in line with a previous investigation (Parai et al., 2018) that demonstrated stable integration of reserpine into the binding pocket of MvfR. Gómez-Gómez et al. (2019) and Shah et al. (2019) reported that the docking study of the AgNP with MvfR showed acceptor interaction with Asn206, Leu207, and Arg209, which advocated the interaction of SeNP and MvfR in the current study. To summarize, the computational analysis revealed that SeNPs established favorable interactions with mvfR, potentially suppressing the PQS signaling in *P. aeruginosa* infections. Consequently, SeNPs hold promise as potential agents for combating drug-resistant biofilm formation associated with *P. aeruginosa*. The current molecular-docking investigations (Figures 1, 2; Figures 7–9) reveal unequivocally that selenium strongly locks within the catalytic groove of the active domain of the AHL synthase (LasI/RhlI) and receptor proteins (LasR/RhlR), thereby inhibiting the expression and signaling of QS-controlled virulence genes and halting the formation of biofilm in *P. aeruginosa*.

The established mechanism of action of SeNPs is not reported (Singh et al., 2017; Gómez-Gómez et al., 2019; Shah et al., 2019) and is warranted to be defined by *in vitro* and *in vivo* experiments. In addition, SeNPs does not bear chemical structural similarity to AHLs and SAM counterparts in QS-regulated biofilm formation, suggesting a novel approach to inhibiting QS-controlled genes and a possible treatment option for *P. aeruginosa*. A conclusive statement about their mechanism of action may be currently available in the present study. However, the mode of action for other nanoparticles have been hypothesized and presented by other scientists (Yin et al., 2020). For instance, AgNPs are effective antimicrobial agents that produce silver ions responsible for the inhibition of bacterial enzymatic systems including DNA synthesis (Wang et al., 2018). Many mechanisms have been postulated to underlie silver antimicrobial action, including cell wall and cytoplasmic membrane disruption, inhibition of protein synthesis machinery by denaturation of ribosomes, interference with ATP production and chemiosmosis, production of ROS, and interference with DNA replication machinery (Yin et al., 2020). Therefore, on the basis of the hypothesis about the mechanism of action of AgNPs, we foresee that SeNPs can serve as potential monotherapeutic agents or conjugates with available drugs, in order to enhance their efficacy and biocompatibility with reduced toxicity. In the current study, we examined the potential of SeNPs. Selenium is an essential nutrient, used for dietary consumption required for human metabolism. Thus, it is safe for use at nanoscale (Gunti et al., 2019). In addition, smaller size and high volume-surface area make the nanoparticles enhanced substitutes to increase the penetration to microbial cells for action and also to develop a drug delivery system (Singh et al., 2017).

Two researchers suggested that glutamine, histidine, serine, threonine, and tyrosine significantly enhanced growth and biofilm formation (Velmourougane and Prasanna, 2017). Other studies also suggested that asparagine, histidine, leucine, methionine, tryptophan, and tyrosine are involved in the architecture of biofilm assembly (Kolodkin-Gal et al., 2010). Our findings showed interactions of SeNPs with key amino acids residues, such as glutamine, histidine, threonine, and tyrosine (Table3), in ligand binding or active domains of the QS signaling proteins. Moreover, selenium (Se) is more reactive towards electrophiles due to their lower electronegativity and nucleophilic behavior; thus, it is a hydrogen (H) bond acceptor (Goldsztejn et al., 2022) that supports our data (Table 3). In addition, the PDB survey also suggested the occurrence of strong Se–H bonding.

The active site residues of examined proteins (Table 2) are involved in the interaction with SeNPs, where amino acid residues show strong hydrogen and hydrophobic bond formation desolvation energy or atomic contact energy or binding energy (kcal mol^-1^) represented in Table 3. Generally, these binding energies reflect the mirror of Gibbs-free energy, which describe the efficiency of any reaction as well as binding or interaction of the residues. Negative value (−) of binding energy represents higher affinity of the residues resulting in strong interactions. To the best of our knowledge, unavailability of the co-ordinate or geometric confirmation file of SeNPs to support any available force field narrowed down the possibilities to perform post-docking simulation for further energy calculations.

In the present study, our analysis reveals the potential of SeNPs that can be used as an anti-QS agent monotherapeutically or as a conjugate with available therapeutics to increase efficacy. We examined the active binding sites (Table 2) of the targeted protein, and all the interactions in the present study revealed that SeNPs could bind at the active domain of the examined protein.

**FIGURE 10:**
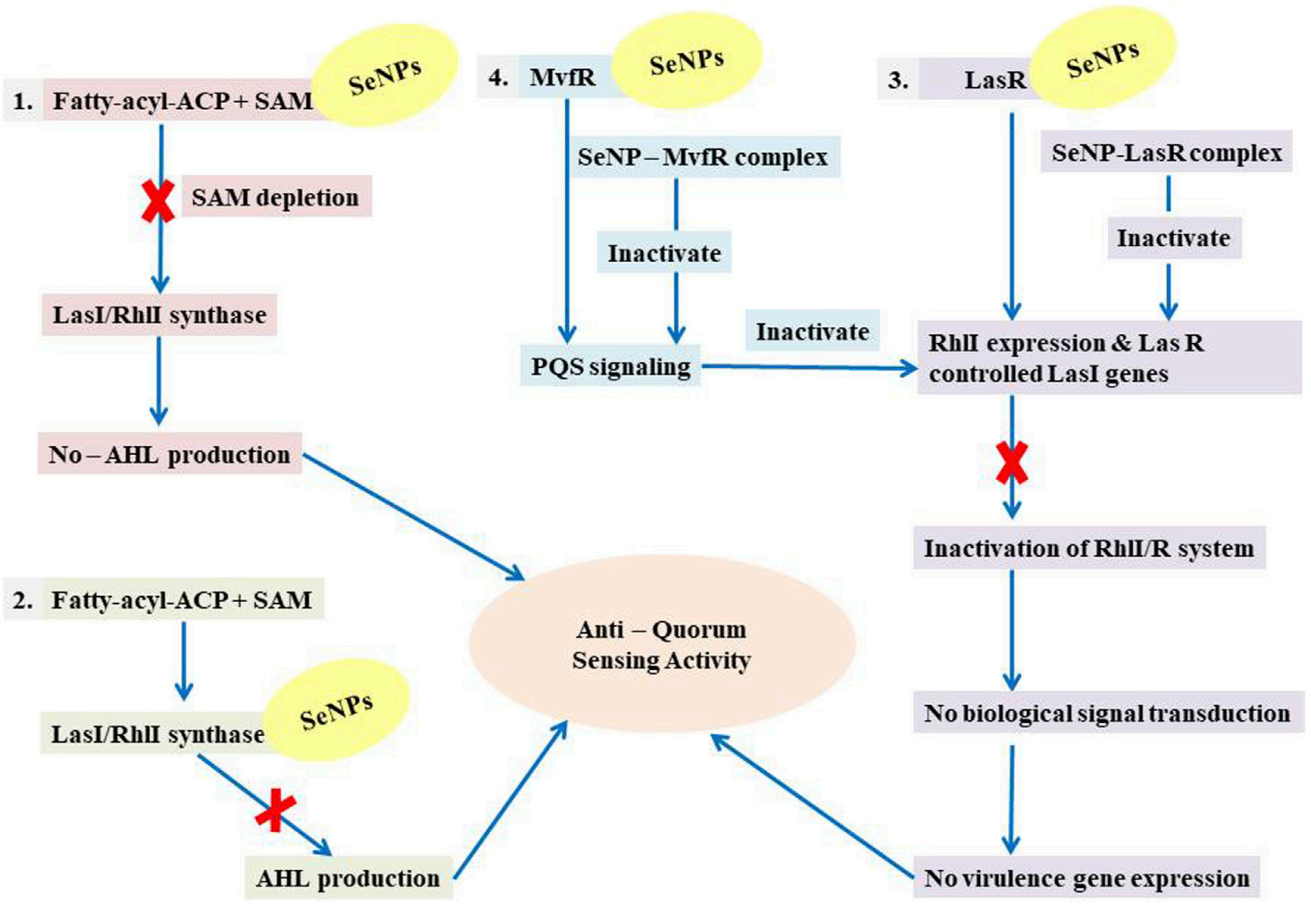
Illustration of the mechanistic pathway of QS inhibition.

LasI is the counterpart of the AHL synthase. Biochemical studies, both *in vitro* and *in vivo*, demonstrated that enzymes in the LuxI family (for example, LasI) used S-adenosyl-metheonine (SAM) and Acyl-acyl-carrier protein (Acyl-ACP) as a substrate to produce AHL molecules which stimulate the QS towards biofilm formation as well as adaptive mode of drug resistance. In the current study, SeNPs interacts with LasI residues, Phe105, which belongs to the counterpart residues of the AHL synthase, and Thr121 which is a binding site of SAM with AHL synthase to generate a signal and produce AHL molecules (Gould et al., 2004; Lidor et al., 2015; Maisuria et al., 2016). Therefore, these findings lead to an understanding that SeNPs can also competitively bind with SAM and AHL synthase active sites to suppress the stimulus of QS signals (Figure 10; Table 3). The LasI synthase receptor protein, LasR, has two functional domains, namely, 1) ligand-binding domain (LBD) and 2) DNA-binding domain. Zou and Nair (2009) established that the LBD of LasR contains an L3 loop (active binding pocket) with residue length Leu40 to Phe51 (Zou and Nair, 2009). In addition, Paczkowski and coworkers (2019) demonstrated that the LasR binding pocket (Leu40-Phe51) displays significant flexibility in accommodating different ligands (Paczkowski et al., 2019). Therefore, in our study, SeNPs are known to interact with Lys42 of the LasR-LBD (L3 loop) through hydrophobic interaction and advocate the inhibitory potential of SeNPs against AHL production to stop QS in *P. aeruginosa* (Figure 10). A research group showed that the LBD of RhlI is made up of an activation, allosteric, and inhibition pocket including Leu102 and V138 and explained as a potential target to design an antagonist molecule (Shin et al., 2019), which supports our study (Table 3). Similarly, Simanek and team (2022) demonstrated that pyocyanin production, a virulence factor of biofilm, is controlled through physical interaction between RhlR and PQSE, the last gene in the operon of PQS synthesis (Simanek et al., 2022). Another research study showed the straight involvement of PQS in rhamnolipid synthesis (Reis et al., 2011). Moreover, a team of researchers (Ilangovan et al., 2013) showed the functional ligand-binding domain of MvfR containing a hydrophobic pocket with residues of Leu208 and Arg209. Our results corroborate with the previously published literature (Ilangovan et al., 2013) and found that SeNPs interacted with Leu208 (hydrogen bond) and Arg209 (hydrophobic bond) and justify its inhibitory capacity to obstruct the PQS molecules resulting in the inactivation of the RhlI/R QS system (Figure 10). Overall, our finding points towards the understanding that particular protein residues are strongly bonded with SeNPs and can act potentially toward imparting the transcriptional signals resulting in the suppression of virulence factors and downregulation of biofilm formation.

Moreover, we show for the initial moment that SeNPs can attach to the signal synthases LasI (Figure 1) and RhlI (Figure 7), despite the fact that their interaction and binding have not previously been documented in the literature. We proposed several possible strategies by which SeNPs disrupt QS networks based on our *in silico* assessments as follows: 1) Inhibition of S-adenosyl methionine (SAM) biosynthesis: SAM functions as an amino donor in the synthesis of the homoserine lactone ring moiety. The manufacture of SAM must be inhibited in order to stop AHL formation. It has been speculated that SeNPs attach to SAM, depleting SAM levels and preventing the production of AHL (Figure 10). 2) Inhibition of LasI/RhlI synthase: Inhibition of signal biosynthesis (LasI/RhlI synthase) can be achieved through SeNPs binding to AHL synthase, ultimately hindering the enzymatic activity. Consequently, if AHL production ceases, QS will not occur, as illustrated in Figure 10. 3) Signal receptor proteins LasR and RhlR can be interfered with: Once the bacterial cell density reaches a specific threshold, the LasI/R QS system is triggered. Se binds to LasR’s active site, forming the Se-LasR complex, which then suppresses RhlI expression as well as LasR-controlled genes, including LasI signal synthase. As a result, the RhlI/R system is deactivated, leading to the inhibition of QS-controlled virulence genes expression, as shown in Figure 10. 4) Interference with *Pseudomonas* quinolone signaling proteins MvfR: Se binds to MvfR’s active site, resulting in the Se-MvfR complex, which suppresses MvfR and RhlR controlled gene expression, leading to the deactivation of the MvfR/RhlR system. Consequently, the transcription of QS-regulated virulence genes is blocked and inhibited, as depicted in Figure 10. This study reveals the inhibitory potential of SeNPs using multiple bioinformatics tools. As a result, they may facilitate the development and implementation of such compounds to increase anti-quorum sensing reactions during *P. aeruginosa* infection.

## 4 Conclusion

The link between QS and bacterial pathogenicity is almost universally acknowledged. Interference bacterial cell-to-cell communication has been determined to be a promising technique for developing novel antimicrobial, anti-QS, and chemotherapeutic drugs. This opens the way for new antivirulence chemicals to be developed that are both effective and efficient. Inhibition of QS has the potential to reduce virulence. A similar conclusion was found about the anti-QS activity of selenium nanoparticles using computational investigation in the present study. The docking results demonstrated that SeNPs bind to the active sites of LasI/R, RhlI/R, and MvfR with consistent binding energies, suggesting that they could be used as an antivirulence agent against *P. aeruginosa* QS signaling in the future. Surprisingly, selenium nanoparticles bear no chemical structural similarities to AHL counterparts, suggesting a novel approach to inhibiting QS-controlled genes and a possible treatment option for *P. aeruginosa* infections. The interaction of selenium nanoparticles with the AHL synthase (LasI/RhlI) may interfere with the production of AHL, which may impede QS signaling. By attaching to receptor proteins (LasR/RhlR and MvfR), they also promise to suppress transcriptional activator activity. The molecular docking investigation found that Selenium nanoparticles were coupled to the active sites of the proteins LasI (Phe105 and Thr121)/RhlI (Leu102 and val138), LasR (Lys42, Arg122, and Glu124)/RhlR (Tyr43, Tyr45, and His61), and MvfR (Leu208 and Arg209). Consequently, nanoparticle-based attenuation of QS in *P. aeruginosa* can be a countermeasure substitute to conventional antibiotics for the therapeutic management of these bacterial infections. Overall, the *in silico* results demonstrate that SeNPs can prevent bacterial infections and may be a potential target for suppressing *P. aeruginosa* QS-mediated pathogenicity and can be designed in several ways to combat bacterial virulence at a feasible cost. However, these anti-QS medications require more in-depth studies before they can be administered in patients. In addition, gene expression analysis along with *in vitro* and *in vivo* experiments such as biocompatibility, cell toxicity, and clinical investigations are prospective research questions which needs to be probed and studied, to determine the efficacy and dosage of SeNPs, before administering in *P. aeruginosa*-infected persons.

## Data Availability

The original contributions presented in the study are included in the article; further inquiries can be directed to the corresponding author.
